# Comparative prospective randomized study of minimally invasive transpiriformis approach versus conventional posterolateral approach in total hip arthroplasty as measured by biology markers

**DOI:** 10.1007/s00264-021-05083-5

**Published:** 2021-05-27

**Authors:** Cong Xiao, Zhixiang Gao, Shaoyun Zhang, Nengji Long, Kai Yao, Peng Cai, Fenglai He, Lijuan Liu, Yishan Jiang

**Affiliations:** Department of Orthopedics, The Third Hospital of Mianyang, Sichuan Mental Health Center, No. 190 The East Jiannan Road, Mianyang, 621000 China

**Keywords:** Total hip arthroplasty, Piriformis, Posterolateral, Posterolateral approach, Short external rotators

## Abstract

**Background:**

Minimally invasive surgery is becoming increasingly common, but evidence of the advantages of the minimally invasive transpiriformis approach in total hip arthroplasty is limited. Therefore, our single-centre randomized controlled trial evaluated the benefits of this approach versus the posterolateral approach.

**Methods:**

Forty-nine patients undergoing the minimally invasive transpiriformis approach and 57 patients undergoing the posterolateral approach total hip arthroplasty were analyzed. The operative time, length of hospital stay, incision length, and peri-operative data related to the surgery were recorded. In both groups, serum CRP, IL-6, HGb, Hct, MB, CK-MB, and CK levels, Harris hip scores, and VAS scores were recorded.

**Results:**

Patients who underwent the minimally invasive transpiriformis approach had a smaller surgical incision (9.10 ± 0.94 vs. 15.56 ± 1.20 cm, P = 0.00) and shorter hospital stay (6.20 ± 1.54 vs. 12.26 ± 2.97 days, P = 0.00) than those who underwent the posterolateral approach. Serum levels of CRP, IL-6, MB, CK-MB, and CK were also lower. According to the Harris hip score, the minimally invasive transpiriformis group showed significant improvement at one week and one month after surgery.

**Conclusion:**

Compared to the posterolateral approach, the minimally invasive transpiriformis approach for total hip arthroplasty provided rapid functional recovery, elicited a significantly reduced post-operative inflammatory response, and caused less muscle damage.

## Introduction

Since the early 1960s [1], when the father of modern hip replacement, John Charnley, performed joint replacement surgery, artificial joint replacement has evolved and is now a mature, standard orthopaedic treatment technique that can effectively restore joint function and improve the quality of life of patients [2, 3]. However, the disadvantages of traditional total hip arthroplasty (THA) with the posterolateral approach (PLA), such as greater trauma, more blood loss, significant pain, and a high dislocation rate [4], have led to increasing concerns regarding how to minimize the intra-operative damage to the soft tissues of the hip, which poses a new challenge to orthopaedic surgeons. Minimally invasive THA has grown in popularity worldwide during the last decade due to its smaller skin incisions, reduced muscle damage, shorter post-operative hospital stays, and faster functional recovery [5, 6]. Currently, the surgical approaches for minimally invasive THA include anterior, anterolateral [7, 8], lateral [9], posterolateral, and posterolateral approaches [10], each of which has its own advantages and disadvantages. Although anterior and anterolateral surgery can be achieved through the intermuscular plane, which preserves the posterior joint capsule and reduces the risk of post-operative joint dislocation, there are difficulties in terms of femoral exposure or even fracture, the high error rate of prosthesis implantation, risk of vascular nerve injury, steep learning curve, and need for custom-made instruments [11, 12].

In 2008, Penenberg et al. [13]. proposed percutaneously assisted total hip arthroplasty (PATH) without disruption of the external rotator muscle group; however, customized instrumentation is required to assist in the implantation of the prosthetic acetabular cup. Therefore, in 2012, Roger et al. [14] suggested performing THA through the transpiriformis approach based on Penenberg et al. In 135 patients, the average surgical incision length was 9 cm, with no dislocation, no sciatic nerve palsy, no wound complications, a low transfusion rate (8%), an average acetabular cup abduction angle of 41° (range 21–49°), an average anteversion angle of 21° (15–27°), femoral prosthesis with more than 2° of varus deviation in 4%, and more than 2° of valgus deviation in 2%, with good clinical outcomes. However, the study was retrospective and lacked comparisons with other surgical approaches. Therefore, our goal was to determine whether this approach offers superior clinical results by a prospective randomized controlled trial.

The definition of a minimally invasive total hip is not limited to an incision of less than 10 cm but also little damage to soft tissues and muscles [9, 15]. Studies have shown that elevated post-operative serum creatine kinase (CK) can reflect the degree of muscle damage and that elevated C-reactive protein (CRP) can reflect the degree of inflammation, so the traumatic effects of surgery can be measured objectively using CK and markers of inflammation [16, 17]. To date, no relevant studies in the literature have reported changes in regard to muscle injury and inflammatory indexes in minimally invasive THA through the transpiriformis approach. We hypothesize that minimally invasive THA through the transpiriformis approach can reduce post-operative CK and CRP indexes and reduce pain in patients.

## Materials and methods

**Fig. 1:**
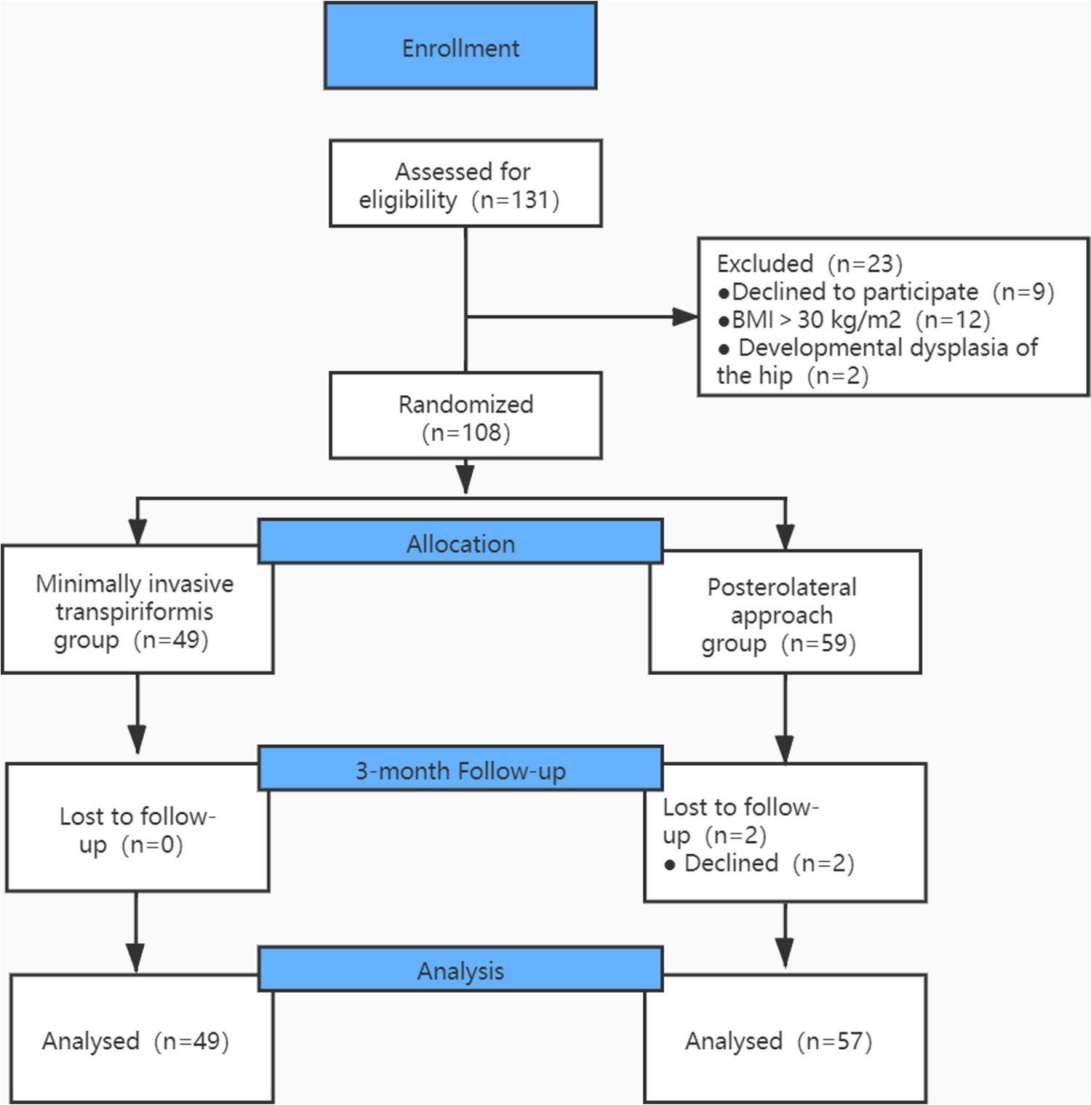
Patient flow chart

This study is a prospective, randomized controlled trial approved by the ethics committee of the hospital and registered with the Chinese Clinical Trials Registry (ChiCTR2000029515). A total of 106 patients diagnosed with unilateral femoral neck fracture between December 2019 and May 2020 were recruited for this study, and all patients provided informed written consent (Fig. 1).

Inclusion criteria are as follows: unilateral primary or secondary hip osteoarthritis, femoral neck fracture, and body mass index < 30 kg/m^2^. Exclusion criteria are as follows: hip ankylosis, hip flexion contracture, developmental dysplasia of the hip, previous history of hip surgery, bone cement prosthesis, neurological disorders, or musculoskeletal impairments that adversely affect gait or weight-bearing and refusal to participate in the study. Simple randomization was performed using a random number table randomization to assign patients to either the minimally invasive transpiriformis approach group or the posterolateral approach group. During the inclusion period (December 2019 and May 2020), there were 131 primary total hip arthroplasties performed at our hospital. Nine patients declined to participate, 12 patients had a body mass index (BMI) over 30 kg/m^2^, and two patients had developmental dysplasia of the hip. Thus, 108 patients were eligible for randomization. Forty-nine were randomly assigned to the minimally invasive transpiriformis approach, and 59 were randomly assigned to the posterolateral approach. The follow-up of all patients was three months. Zero patients were lost to the minimally invasive transpiriformis approach, and two patients were lost to the posterolateral approach group, which resulted in 49 patients in the minimally invasive transpiriformis approach and 57 patients in the posterolateral approach.

Baseline demographic data including sex, age, weight, height, body mass index (BMI), American Society of Anesthesiologists (ASA) grade, pre-operative myoglobin (MB), CK, CRP, interleukin 6 (IL-6), haemoglobin (HGb), and haematocrit (Hct) levels were recorded for all patients before surgery. The pre-operative general data of the patients in both groups are presented in Table 1, and the differences between the groups were not statistically significant.

**Table 1:** Pre-operative characteristics of patients

	Minimally invasive transpiriformis approach (n = 49)	Posterolateral approach (n = 57)	Difference in means(95%CI)	P value
Age (years)	71.06 ± 10.87	73.93 ± 10.02	2.03 (− 6.89–1.16)	0.16
Sex (M/F)	16/33	26/31	-	0.23
BMI (kg/m^2^)	26.73 ± 4.18	26.39 ± 4.64	0.86 (− 1.37–2.05)	0.69
ASA grade	2.01 ± 0.12	2.05 ± 0.39	0.06 (− 0.16–0.06)	0.36
Side (L/R)	21/28	24/33	-	0.94
CRP (mg/l)	16.83 ± 12.77	17.70 ± 14.40	2.66 (− 6.15–4.41)	0.74
IL-6 (pg/ml)	4.67 ± 1.87	4.84 ± 1.22	0.30 (− 0.77–0.42)	0.56
CK (U/L)	79.25 ± 13.01	77.53 ± 27.53	4.29 (− 6.79–10.24)	0.69
CK-MB (U/L)	8.41 ± 5.68	9.55 ± 4.84	1.02 (− 3.17–0.88)	0.27
MB (ng/ml)	51.88 ± 11.34	53.89 ± 7.40	1.84 (− 5.65–1.64)	0.28
HGB (g/l)	121.58 ± 11.24	117.32 ± 17.45	2.90 (− 1.49–10.02)	0.15
HCT (%)	37.15 ± 3.50	36.32 ± 4.04	0.74 (− 0.64–2.29)	0.27

We recorded the surgery time, skin incision length, surgery time, blood transfusion rate, and hospital stay. The changes in the levels of CRP, IL-6, HGb, Hct, MB, CK-MB, and CK on post-operative days one and three were recorded. Assessment of pain on post-operative days, zero, one, two and four was performed using the visual analog scale (VAS), with a score of 0 indicating no pain, 10 indicating severe pain, and 0 to 10 indicating varying degrees of pain. Additionally, the Harris hip score (HSS) was measured at one week, one month, and three months post-operatively.

### Surgical approaches

#### Minimally invasive transpiriformis approach

**Fig. 2:**
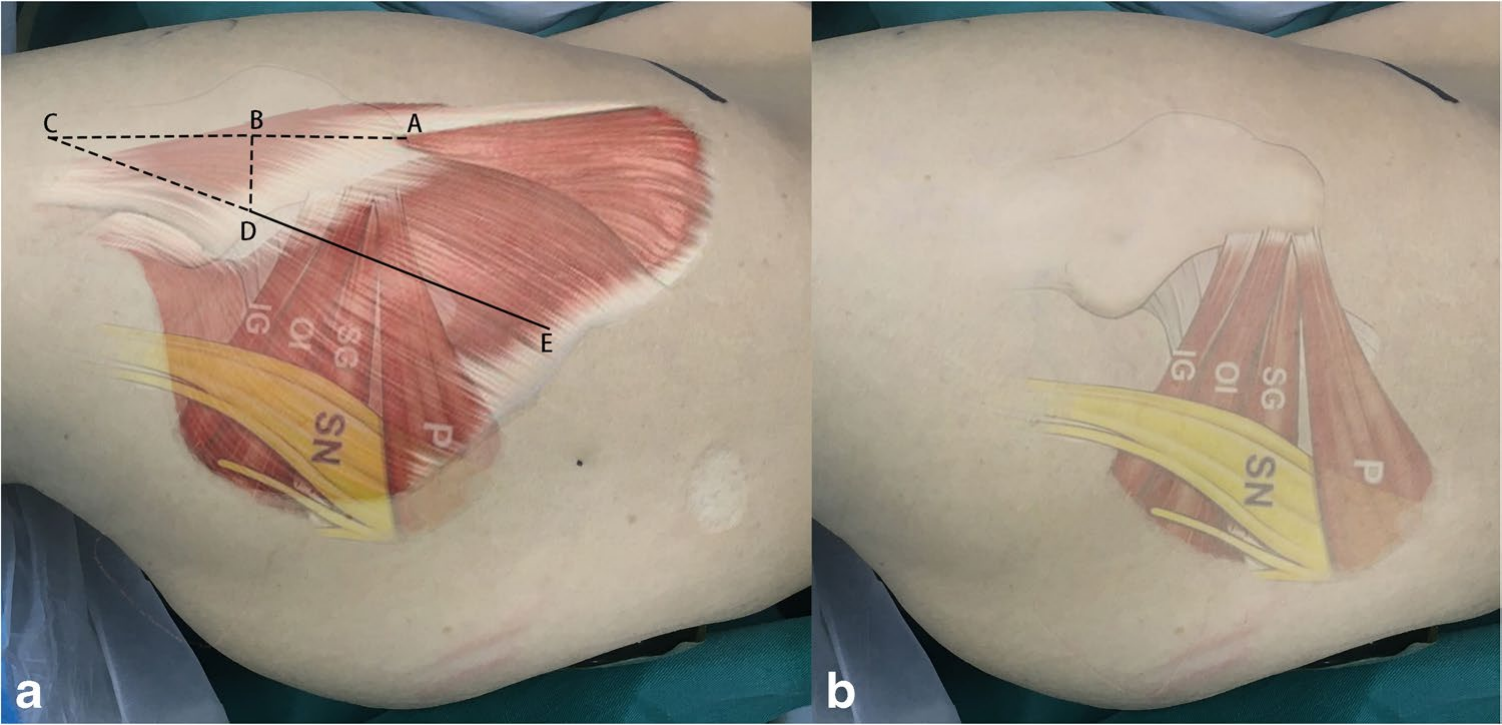
**a** The patient is positioned laterally on the operating table, and the incision is approximately 9 cm DE. **b** The gluteus maximus muscle is split in the direction of the gluteus maximus fibers to reveal the short external rotators such as the musculus piriformis. P, “piriformis”; SG, “superior gemellus”; OI, “obturator internus”; IG, “inferior gemellus”; SN, “sciatic nerve”

**Fig. 3:**
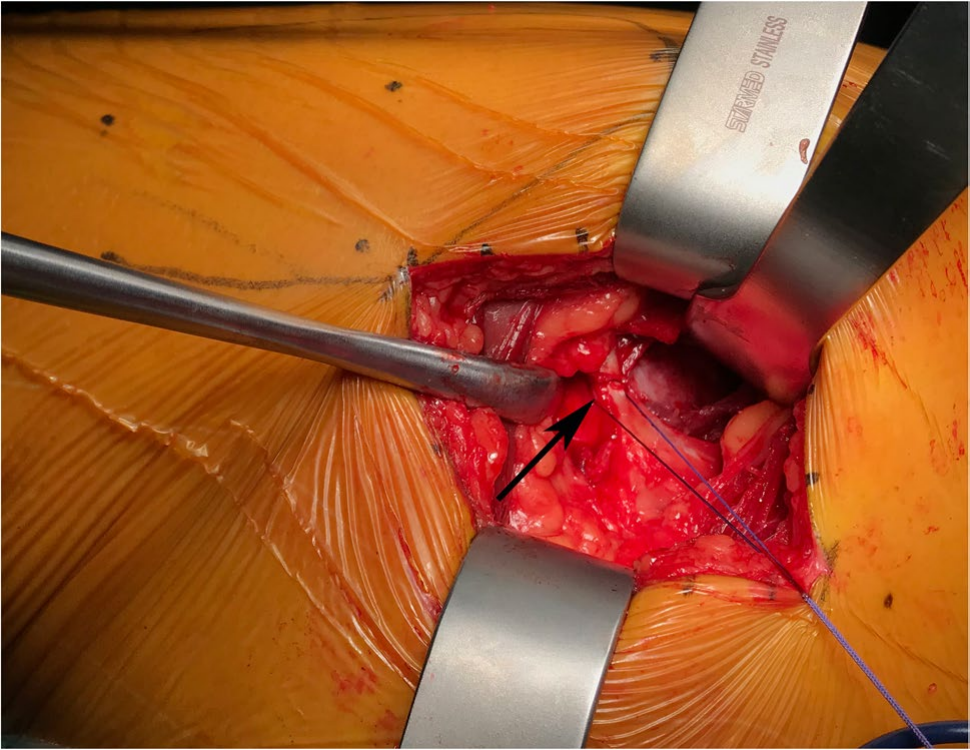
Confirmation and suturing of the piriformis tendon, the release of the tendon at the greater trochanter. The black arrow is the piriformis tendon

**Fig. 4:**
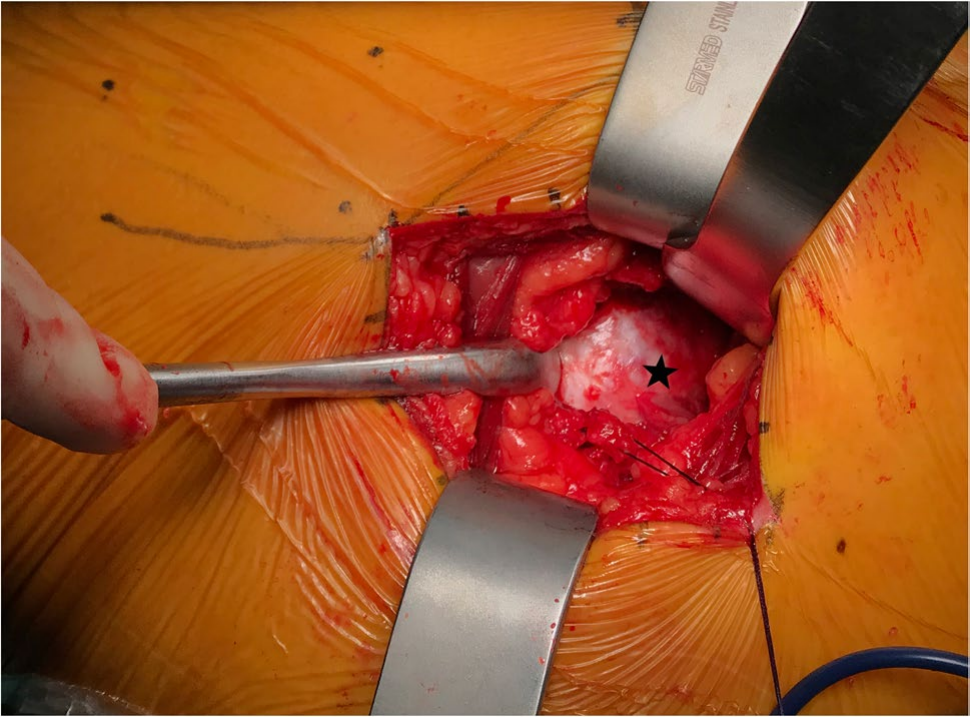
The joint capsule is visible after dissection of the piriformis tendon. The black pentagon is the posterior joint capsule

**Fig. 5:**
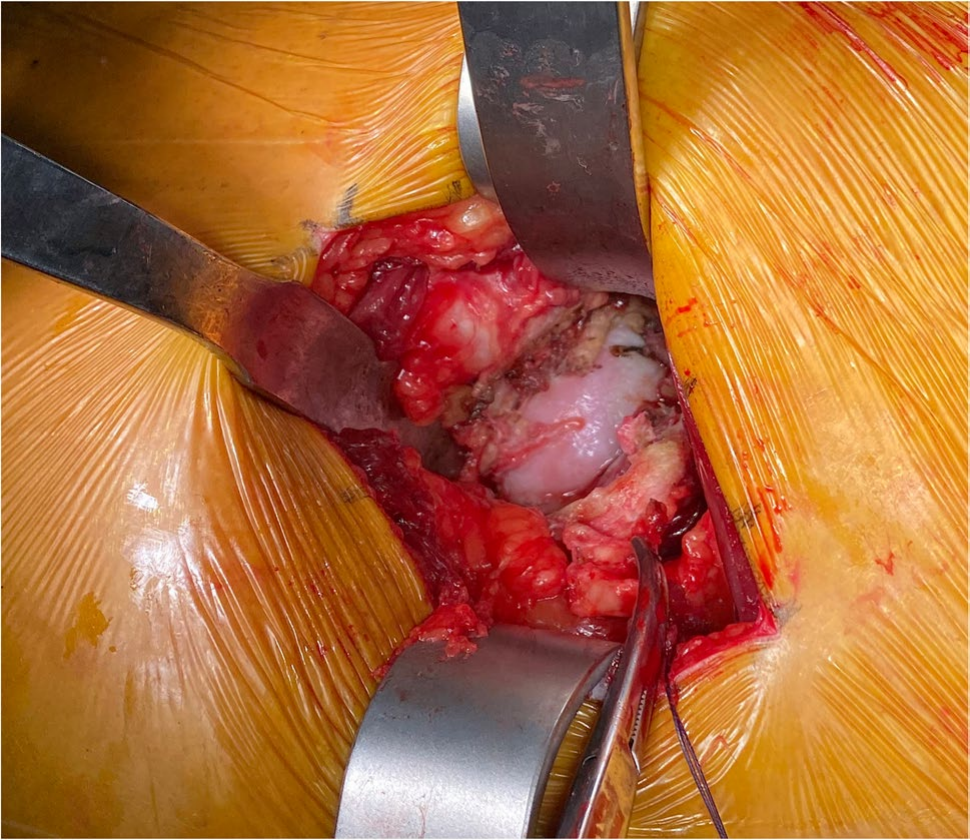
Two Hoffman hooks were placed to expose the femoral neck

An experienced orthopaedic surgeon carried out all operations. The patient is placed in the lateral position. Take the greater trochanter of the femur as the starting point along the direction of the femoral stem marked AB = 4 cm and BC = 5 cm, respectively, and draw BD = 3 cm perpendicular to AC, with point D as the starting point extending proximally along the direction of CD for an average of DE = 9 cm as the surgical incision (Fig. 2a). The skin and subcutaneous tissues were incised, the gluteus maximus fibers were bluntly separated along the gluteus, and the deep pyriform muscle was explored (Fig. 2b). Hoffman’s pull hooks were placed superior and inferior to the piriformis muscle to protect the gluteus medius and the external rotators (internal obturator muscle and gemellus superior, respectively) (Fig. 3). The posterior joint capsule was exposed by cutting the stop of the pyriform muscle on the greater trochanter and scalloping the joint capsule to expose the femoral neck (Figs. 4 and 5).

**Fig. 6:**
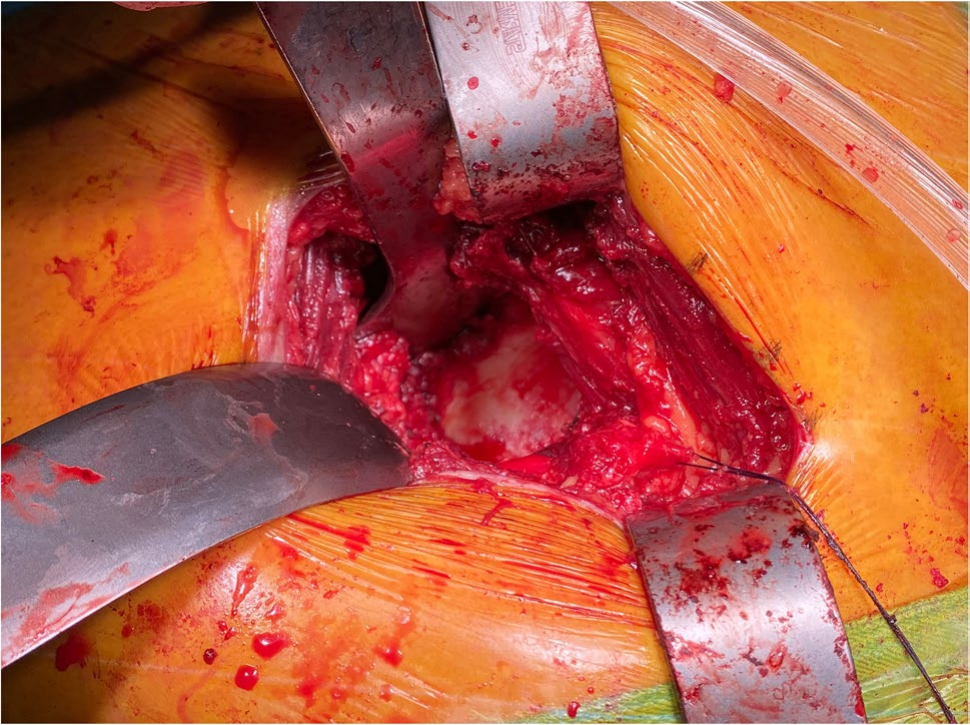
Two Hoffman hooks and two retractors were placed to expose the acetabulum

**Fig. 7:**
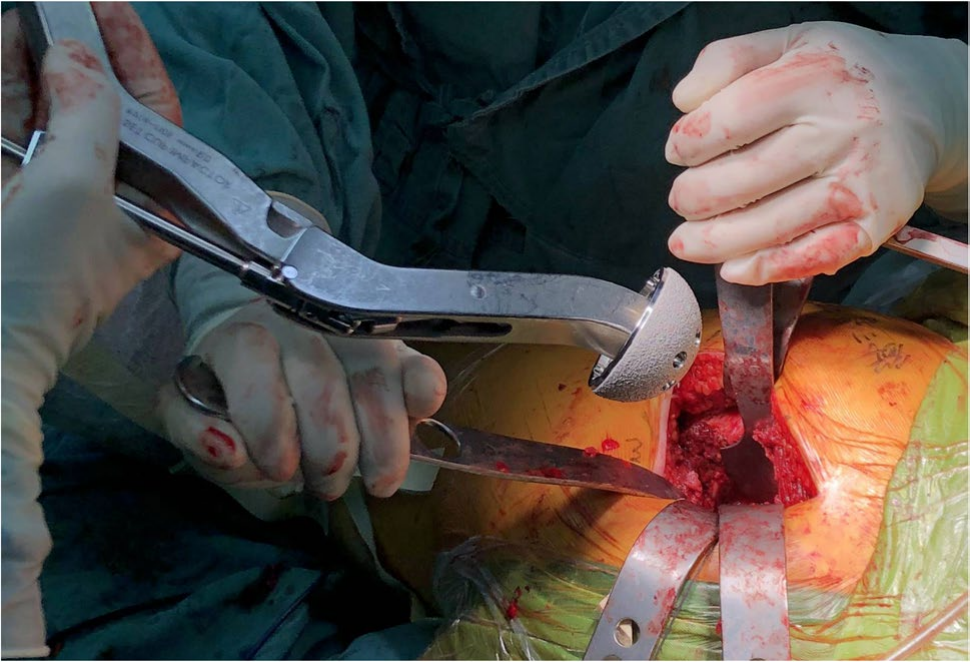
Acetabulum polished by eccentric file and implanted with prosthesis

**Fig. 8:**
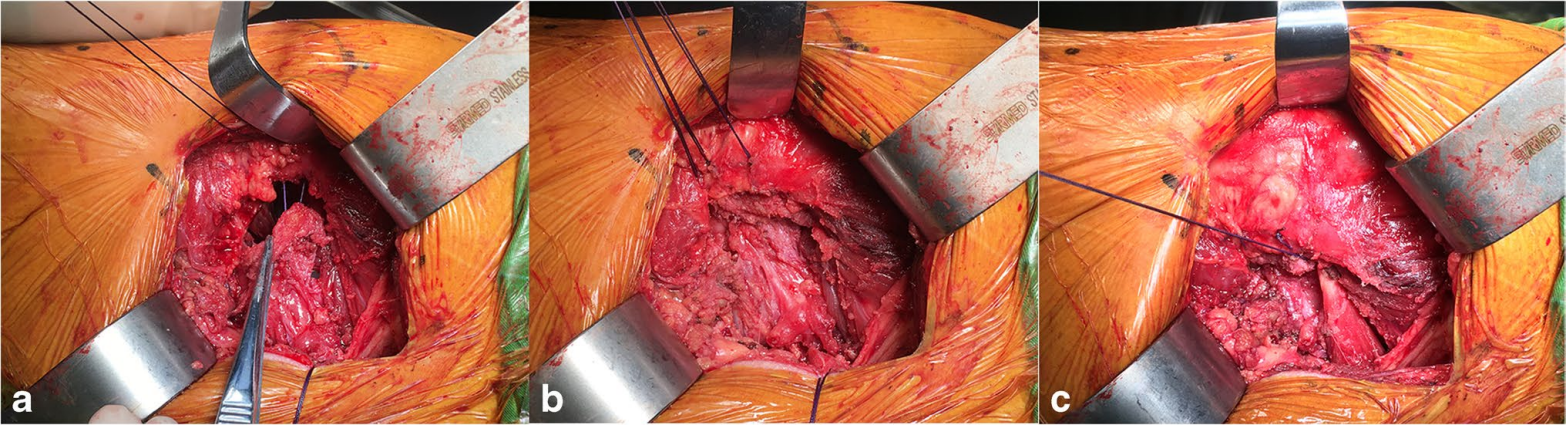
The posterior joint capsule and piriformis tendon were repaired in turn at the greater trochanter

**Fig. 9:**
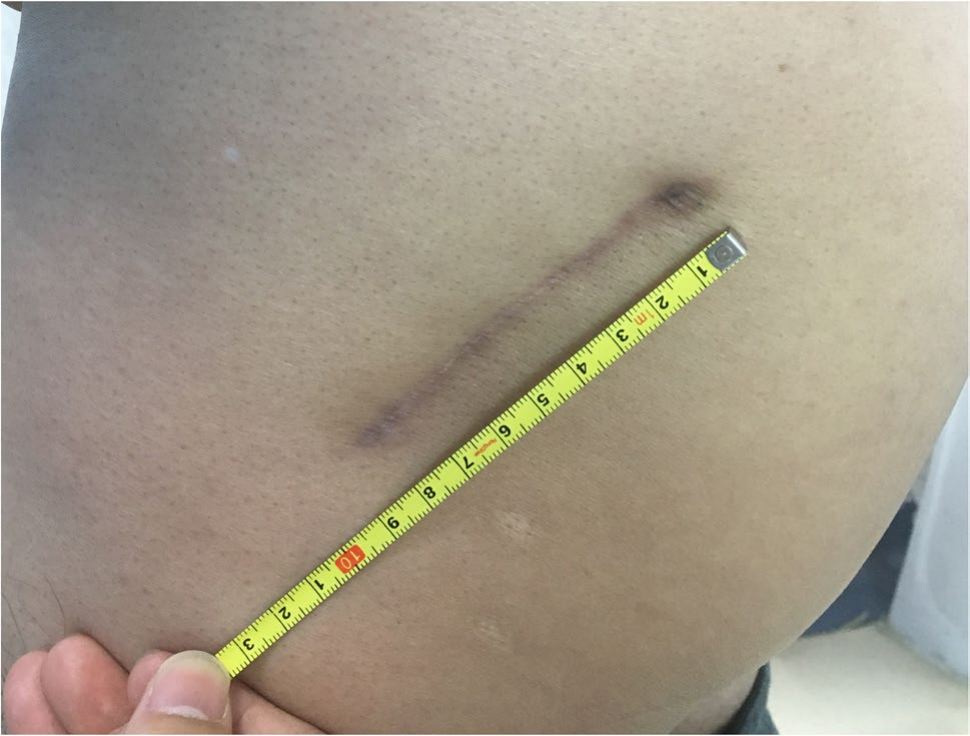
The skin was closed with intradermal sutures

Hip dislocation was achieved by flexion, internal rotation, and internal retraction, and femoral neck osteotomy was performed. Hoffman pulling hooks were placed at each of the anterior and inferior edges of the acetabulum to reveal the acetabulum (Fig. 6). The position of the desired acetabular prosthesis is 40° ± 10° of abduction and 15° ± 10° of anteversion [18] (Fig. 7). The femoral prosthesis was implanted according to preoperative template measurements and intra-operative assessment, and the hip was repositioned by external rotation. The stability of the hip was checked in flexion at 90°, adduction at 20°, and internal rotation at 50°. The wound was extensively irrigated with saline, the joint capsule was repaired, and the pyriform stop was reconstructed; this approach does not require a drainage tube (Fig. 8a–c). The skin was closed with intradermal sutures (Fig. 9).

#### Posterolateral approach (PA)

Patients were placed in the standard lateral position. A surgical incision of approximately 14–16 cm was made on the hip’s posterolateral surface to incise the skin, subcutaneous tissue, gluteus maximus, external rotators, and posterior capsule. For hip joint dislocation, the femoral neck was osteotomized, and the acetabular and femoral prostheses were implanted in the same way as in the transpiriformis approach. The drainage tube was retained post-operatively until the flow was < 50 ml/day.

### Statistical analysis

Comparison of continuous variables between groups was performed by paired t-test and unpaired t-test when necessary; Fisher’s exact t-test or χ^2^ test was applied to compare categorical variables. Spearman’s correlation analysis analyzed the correlation between operative time and CK, with *P* < 0.05 considered significant.

## Results

The minimally invasive transpiriformis group consisted of 49 patients (16 males and 33 females) with a mean age of 71.06 ± 10.87 years; the posterolateral group consisted of 57 patients (26 males and 31 females) with a mean age of 73.93 ± 10.02 years. In the minimally invasive transpiriformis group, the mean operative time was 84.47 ± 19.37 min, and the mean incision length was 9.10 ± 0.94 cm; in the posterolateral group, the mean operative time was 105.44 ± 10.50 min, and the mean incision length was 15.56 ± 1.20 cm. The operative time and incision length were lower in the minimally invasive transpiriformis group (P = 0.00) (Table 2).

**Table 2:** Peri-operative data of patients

	Minimally invasive transpiriformis approach (n = 49)	Posterolateral approach (n = 57)	Difference in means(95%CI)	P value
Length of hospital stay (days)	6.20 ± 1.54	12.26 ± 2.97	0.47 (− 6.99–5.12)	0.00
Length of incision (cm)	9.10 ± 0.94	15.56 ± 1.20	0.21 (− 6.87–6.03)	0.00
Operative time (min)	84.47 ± 19.37	105.44 ± 10.50	2.97 (− 26.86–15.08)	0.00
HGB(g/l)				
1st post-operative day	98.76 ± 10.38	94.09 ± 9.42	1.92 (0.86–8.48)	0.02
1st post-operative day decline rate	18.80 ± 9.15	20.44 ± 10.76	1.96 (− 5.52–2.24)	0.40
3rd post-operative day	92.54 ± 8.89	89.10 ± 1.47	1.19 (1.07–5.81)	0.00
3rd post-operative day decline rate	23.44 ± 10.40	24.05 ± 9.77	1.73 (− 6.10–0.77)	0.13
HCT(%)				
1st post-operative day	30.07 ± 3.06	28.22 ± 0.29	0.41 (1.04–2.66)	0.00
1st post-operative day decline rate	19.12 ± 9.63	21.79 ± 8.19	1.96 (− 4.50–3.28)	0.76
3rd post-operative day	27.83 ± 2.41	25.45 ± 1.27	0.37 (1.65–3.10)	0.00
3rd post-operative day decline rate	24.49 ± 9.54	26.04 ± 1.35	1.28 (− 4.09–0.98)	0.23

Compared with the pre-operative HGb and HCT, the minimally invasive transpiriformis and posterolateral groups decreased gradually on the first and third post-operative days, and the posterolateral group showed a more significant decrease in HGb and HCT after surgery (P = 0.00) (Table 2). HGb and HCT loss were calculated by the difference between pre-operative HGb and HCT and the first and third post-operative days. The minimally invasive transpiriformis group showed a decrease in HGb (18.80 ± 9.15 g/l) and HCT (19.12 ± 9.63%) on the first post-operative day and a decrease in HGb (23.44 ± 10.40 g/l) and HCT (24.49 ± 9.54%) on the third post-operative day. In the posterolateral group, HGb decreased by 20.44 ± 10.76 g/l and HCT decreased by 21.79 ± 8.19% on the first post-operative day, and HGb decreased by 24.05 ± 9.77 g/l and HCT decreased by 26.04 ± 1.35% on the third post-operative day. However, there was no significant difference between the minimally invasive transpiriformis and posterolateral groups in comparing HGb and HCT decline in the first and third days after surgery (P > 0.05) (Table 2).

Serum CRP levels gradually increased on the first and third post-operative days, reaching a maximum on the third post-operative day at our monitoring time point, reaching 97.21 ± 39.27 mg/l in the minimally invasive transpiriformis group and 113.29 ± 4.98 mg/l in the posterolateral group (Table 3). All patients had higher post-operative serum CRP levels in the posterolateral group than in the minimally invasive transpiriformis group; the differences were statistically significant on the first (P = 0.00) and third (P = 0.00) post-operative days (Table 3). Compared to the minimally invasive transpiriformis group, IL-6 levels were significantly higher in the posterolateral group on the first and third post-operative days, with a statistically significant difference (P = 0.00).

**Table 3:** Postoperative inflammation and muscle damage markers of patients

	Minimally invasive transpiriformis approach (n = 49)	Posterolateral approach (n = 57)	Difference in means (95%CI)	P value
1st post-operative day				
CRP(mg/l)	59.51 ± 26.28	69.70 ± 2.95	3.50 (− 17.14–3.25)	0.00
IL-6(pg/ml)	5.33 ± 1.70	6.51 ± 1.75	0.34 (− 1.85–0.52)	0.00
CK(U/L)	476.67 ± 87.44	613.77 ± 25.35	12.13 (− 161.15–113.05)	0.00
CK-MB(U/L)	32.42 ± 21.85	38.41 ± 2.02	2.9 (− 11.75– − 0.22)	0.04
MB(ng/ml)	511.11 ± 189.40	801.09 ± 22.47	25.27 (− 340.10–239.87)	0.00
3rd postoperative day				
CRP(mg/l)	97.21 ± 39.27	113.29 ± 4.98	5.25 (− 26.49–5.68)	0.00
IL-6(pg/ml)	5.66 ± 0.63	6.89 ± 4.13	0.60 (− 2.41–0.05)	0.04
CK(U/L)	370.23 ± 249.37	504.62 ± 21.88	33.15 (− 200.13–68.65)	0.00
CK-MB(U/L)	8.28 ± 5.86	19.20 ± 15.13	2.30 (− 15.48–6.36)	0.00
MB(ng/ml)	148.32 ± 91.98	182.14 ± 12.51	12.30 (− 58.22–9.42)	0.01

**Fig. 10:**
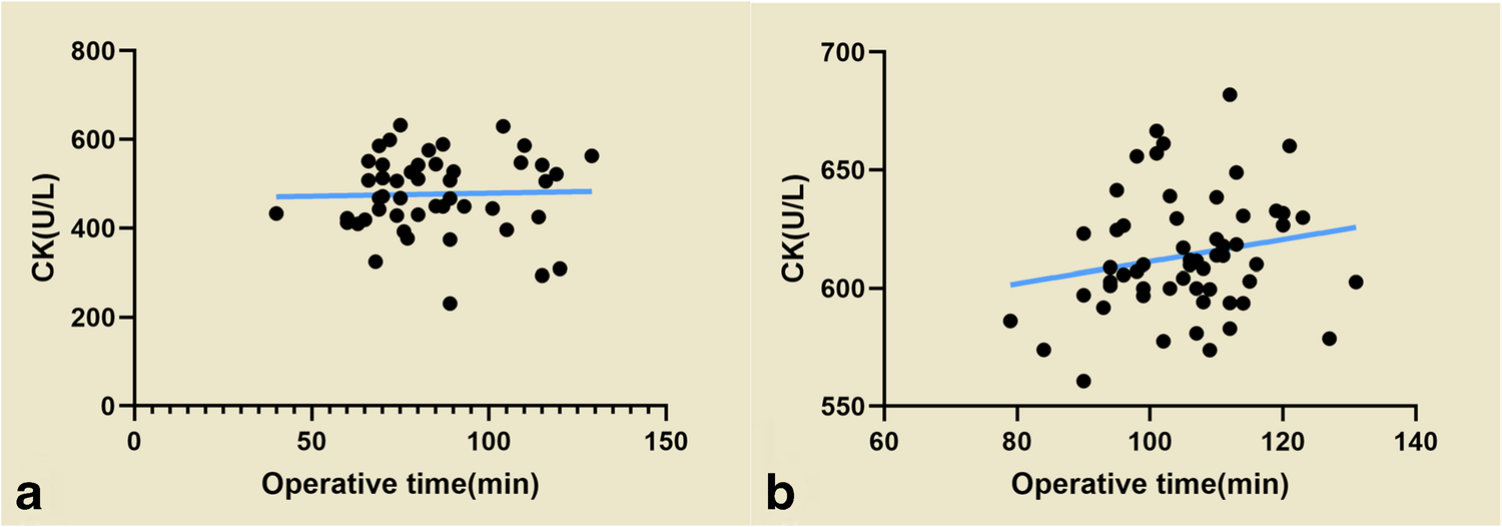
Low correlation between surgery duration and changes in CK levels regardless of whether the patients were in the minimally invasive transpiriformis group (**a**) or the posterolateral group (**b**)

Minimally invasive transpiriformis and posterolateral group serum CK (476.67 ± 87.44 vs. 613.77 ± 25.35) U/L, CK-MB (32.42 ± 21.85 vs. 38.41 ± 2.02) U/L, MB (511.11 ± 189.40 VS 801.09 ± 22.47) ng/ml were elevated on the first post-operative day but showed a decrease on the third post-operative day (CK (370.23 ± 249.37 VS 504.62 ± 21.88) U/L, CK-MB (8.28 ± 5.86 VS 19.20 ± 15.13) U/L, MB (148.32 ± 91.98 VS 182.14 ± 12.51) ng/ml). Overall, the posterolateral group showed higher serum CK, CK-MB, and MB levels than the minimally invasive transpiriformis group (P = 0.00) (Table 3). We next attempted to determine the relationship between surgery time and CK by correlation analysis. Figure 10a and b shows a low correlation between surgery duration and changes in CK levels regardless of whether the patients were in the minimally invasive transpiriformis group or the posterolateral group.

**Fig. 11:**
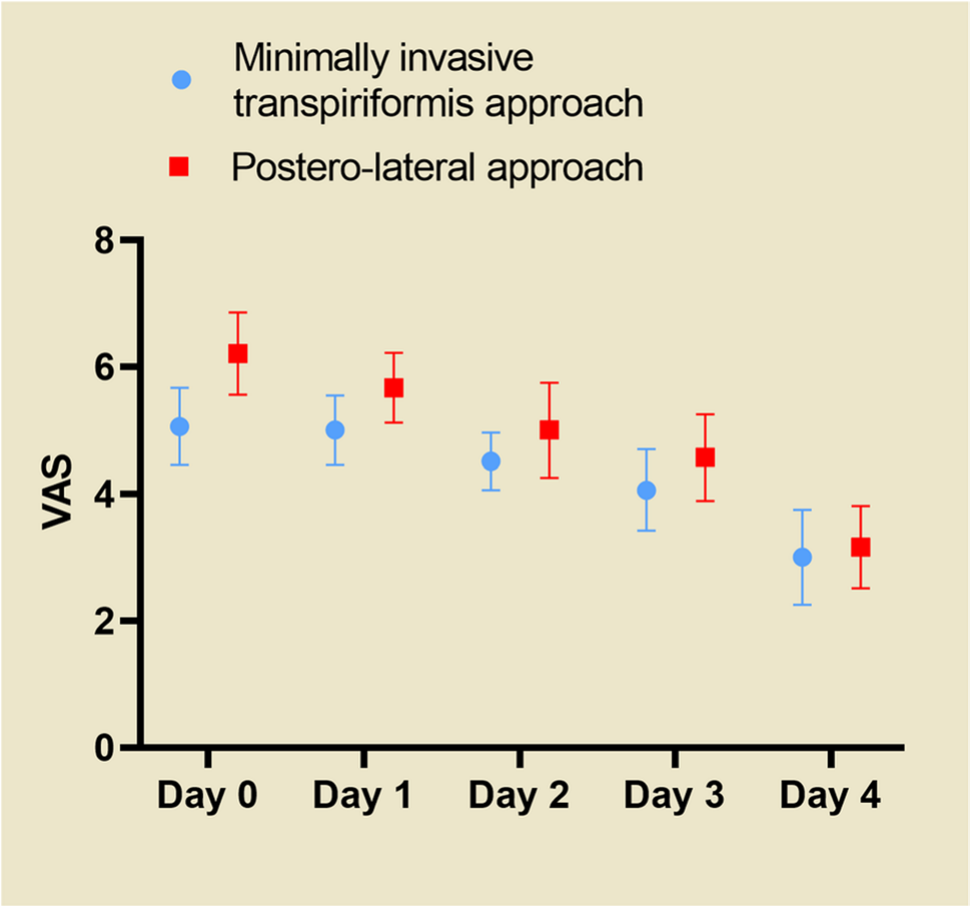
The VAS of the posterolateral approach group was higher than that of the minimally invasive transpiriformis group on the third post-operative day (P < 0.05), and the difference was statistically significant, and there was no statistically significant difference between the two groups on the fourth day of comparison (P > 0.05)

Figure 11 shows that the VAS scores of patients in the posterolateral group were higher than those in the minimally invasive transpiriformis group on post-operative days zero to four, with a statistically significant difference between the two groups observed in the first three days (P = 0.00), but no statistically significant difference was noted on the fourth day (P > 0.05).

**Fig. 12:**
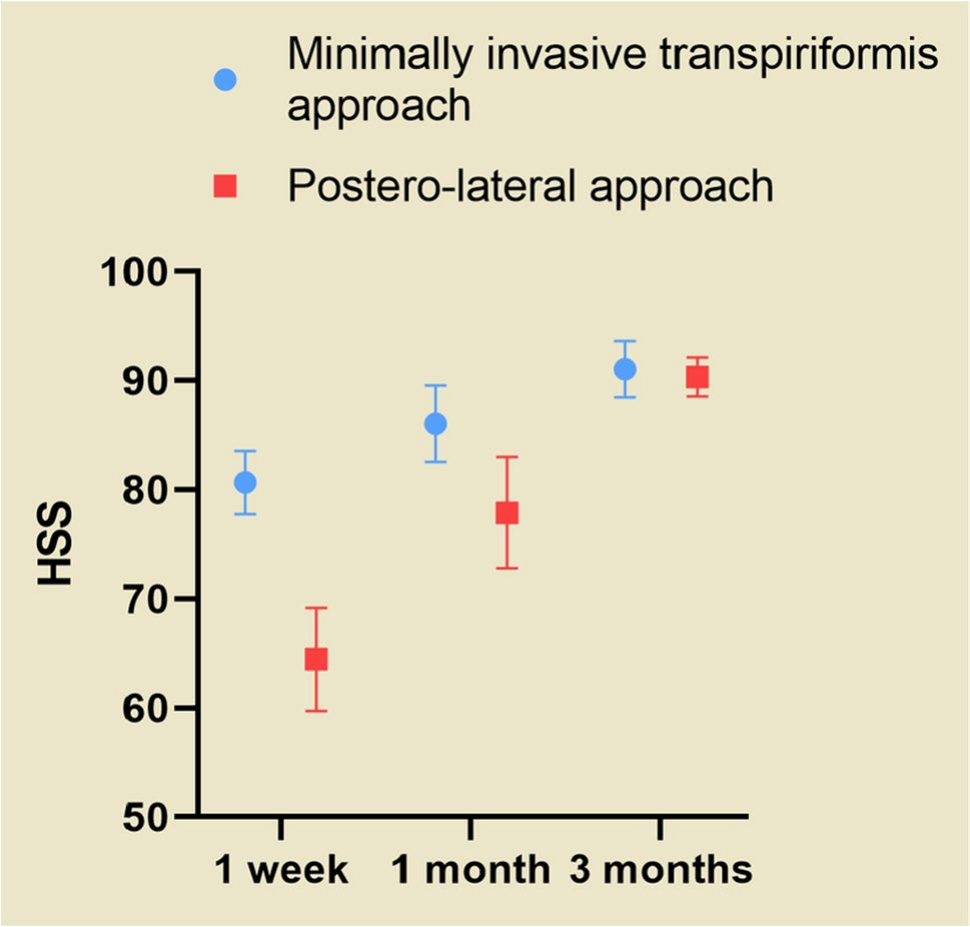
The minimally invasive transpiriformis group had a higher HSS at 1 week and 1 month than the posterolateral group (P = 0.00). The two groups had similar HSS at 3 months after surgery (P > 0.05)

Both groups showed significant improvement in the HSS from pre-operatively to one week, one month, and three months post-operatively. The minimally invasive transpiriformis group had a higher HSS at one week and one month than the posterolateral group (P = 0.00). However, the score of minimally invasive transpiriformis group was only one point higher than that of the posterolateral group at three months post-operatively, and the difference between the two groups was not statistically significant (P > 0.05) (Fig. 12).

## Discussion

Minimally invasive total hip arthroplasty can reduce the damage to the soft tissues around the joint and protect joint stability and power, leading to earlier and better functional exercise tolerance and rapid recovery. Currently, the consensus on minimally invasive total hip arthroplasty is that the surgical incision length should be less than 10 cm [9, 15]. However, this definition is more controversial, and no further measurement of soft tissue damage has been made. Indicators of CK and inflammation best reflect the level of soft tissue damage during surgery. This is an accurate measure of minimal invasion during a surgical procedure.

CK is present in the cardiac muscle, brain, skeletal muscle, and other tissues. Although CK is considered a sensitive marker of myocardial injury, it is present in high concentrations in skeletal muscle, where CK is a dimeric enzyme present in the sarcoplasm. The concentration of CK in the circulation is low in healthy individuals, and when muscle cell membranes are damaged, CK is released into the bloodstream. Therefore, CK is a preferable indicator for assessing muscle damage. Musil et al. [19] compared postoperative levels of CK in a prospective randomized study of patients undergoing the posterolateral approach versus those undergoing the minimally invasive anterolateral (MIS-AL) approach. Serum CK levels among patients undergoing the posterolateral approach were 62.5% higher than those among patients undergoing MIS-AL total hip arthroplasty in the first 48 hours post-operatively. Similar conclusions can be drawn from the current results in the literature [20–23], where most of the patients undergoing minimally invasive THA procedures had with lower CK levels and minor muscle damage. In our study, although pre-operative CK levels were similar in both groups, serum CK levels were lower at day 1 and day 3 after the minimally invasive transpiriformis approach and returned to normal on post-operative day three. Compared with the minimally invasive transpiriformis approach, the incision for THA with the conventional posterolateral approach was longer, averaging 15.56 ± 1.20 cm. In addition to bluntly splitting the gluteus maximus muscle, the traditional posterolateral approach also requires cutting the short external rotators such as the piriformis, internal obturator muscle, and gemellus superior. As a result, muscle damage is serious, and postoperative serum CK levels are high. However, CK levels in serum were reduced in both groups on post-operative day three, which was thought to be related to the half-life of CK. Research suggests that the increase reaches a peak 24–48 hours after muscle injury and then gradually decreases [24, 25]. Bergin et al. [20] showed that the post-operative serum CK levels increased with increasing surgery duration, and similar results were obtained in our study. This also contributes to elevated serum CK levels after the conventional posterolateral approach. The average duration of surgery in the minimally invasive transpiriformis approach group was 84.47 ± 19.37 minutes, and the average time in the posterolateral approach group was 105.44 ± 10.50 minutes. When we analyzed the correlation between operative time and CK by Spearman’s correlation analysis, we found a positive correlation between operative time and post-operative serum CK in both the minimally invasive transpiriformis group and the posterolateral group; however, the correlation was very low (P > 0.05). This may also be related to a type 2 error due to the small number of cases in our study. Naturally, the impact may be due to several factors, such as the proficiency of the surgeon. However, our studies were all performed by the same experienced orthopedic surgeons, so the bias was small. Meanwhile, the transpiriformis surgeries were on average 20 minutes shorter than the standard posterolateral approach. The length of the surgery time is highly related to surgical proficiency. Second, the complexity of the surgical operation is also an important factor in determining the operative time. In the minimally invasive transpiriformis approach, only the joint capsule and piriformis tendon are repaired postoperatively, but in the posterolateral approach, the external rotators (internal obturator muscle and gemellus superior, respectively) is also repaired.

The body elicits a strong stress response during surgery that can cause local damage and organ dysfunction; therefore, it is important for surgeons to minimize the trauma from surgery. CRP is an acute-phase reactant and a sensitive systemic marker of inflammation and tissue injury [26]. However, under stressful conditions such as inflammation, burns, surgery, and trauma, the CRP level increases rapidly within six to eight hours after tissue injury and reaches a peak in 48 ~ 72 hours [27, 28]. The change in serum CRP concentration is positively correlated with the degree of surgical trauma, which is a relatively sensitive indicator reflecting the degree of tissue injury[29]. In our study, post-operative CPR levels were progressively higher in both groups. Moreover, the CRP of patients in the posterolateral group was significantly more elevated than that in the minimally invasive transpiriformis group on either the first or third post-operative day (P = 0.00). The results of one prospective study [20] and three RCTs [19, 21, 23] showed low serum CRP levels in patients after minimally invasive total hip arthroplasty.

Post-operative pain is defined as acute pain associated with an inflammatory process [30], and pain is associated with inflammatory mediators [31]. Proinflammatory cytokines (TNF-a, IL-6) are released from the injury site, which in turn leads to the release of COX-2 and prostaglandin E2 (PGE2) from monocytes and macrophages. One of the causes of postoperative pain is the increased activity of the COX-2 enzyme [32]. On the one hand, COX-2 enzymes play a role in inducing inflammation by increasing the production of proinflammatory prostaglandins in arachidonic acid [33]; on the other hand, COX-2 enzymes increase pain sensitivity in peripheral tissues [34]. We found significantly lower IL-6 levels in the minimally invasive transpiriformis group postoperatively (P = 0.00). The visual analog scale (VAS) was adopted for pain assessment in this study. Jensen et al. [35] showed that a 33% reduction in pain was a reasonable criterion for determining whether the change in pain was meaningful from the patient’s perspective. Therefore, there was no clinical difference when comparing VAS scores between the two groups on the fourth postoperative day in our study. Unfortunately, we did not monitor IL-6 levels in patient serum on the fourth post-operative day to further evaluate the relationship between VAS scores and IL-6. It is also reasonable to assume that the postoperative HSS is associated with pain and muscle damage, and although there was no difference in the VAS score between the two groups on post-operative day four, muscle damage in the posterolateral group severely affected the evaluation of the HSS. Thus, the HSS of patients in the minimally invasive transpiriformis group was significantly higher than that in the posterolateral group at one week after surgery, but this difference gradually decreased as the soft muscle tissues recovered until there was no significant difference in the HSS between the two groups at three months after surgery (Fig. 12).

Several studies have concluded that repair of the posterior joint capsule and short external rotator muscle groups is effective at preventing post-operative dislocation after THA. Relevant clinical studies have concluded that repairing these tissues compared with no repair still resulted in a higher post-operative dislocation rate of 9.5% [36, 37]. In three clinical studies [38, 39, 40], even though the posterior capsule and short external rotators were repaired, they could not withstand the forces generated during tissue healing and failed, with a failure rate of 63–80%. Figures 4 and 5 show that the external rotation muscle groups were well protected in our operative technique, and perhaps, the preservation of these structures may contribute to postoperative hip stability. During the six month post-operative follow-up of all our cases, only one traumatic dislocation occurred. The protection of these structures may improve post-operative gait mechanics, but further studies are needed to clarify this point.

There is still controversy about the body mass index as one of the indications for surgery, and Bostrom et al. [41] suggested that suitable candidates should be patients with a slim body type and a BMI < 30 kg/m2, while minimally invasive surgery is not recommended for initial total hip replacement with too strong muscles and significant anatomical abnormalities. Berger et al. [42] and Roger et al. [14] believed that obesity is a relative rather than an absolute contraindication to minimally invasive surgery; they concluded that although patients with high BMI required a longer skin incision, preservation of the inferior short external was accomplished with exposure and prosthesis implantation.

Samely, we still worry about the skin incision before the surgical operation, so in the inclusion criteria, we choose patients with a slim body type and BMI < 30 kg/m2, to reduce the intra-operative strain on the surgical incision and further avoid wound problems such as skin necrosis and subcutaneous hematoma. None of the minimally invasive groups in our study had wound problems. Therefore, we believe that a lean body type with a BMI < 30 kg/m2 remains one of the indications for minimally invasive surgery. In contrast, the procedure can still be done in patients with strong muscles, obesity, and BMI > 30 kg/m2, but the intra-operative incision needs to be extended, which does not meet the definition of minimally invasive.

In conclusion, in our study, the minimally invasive transpiriformis group had minor muscle damage and a reduced inflammatory response compared to the posterolateral group. Although the minimally invasive transpiriformis approach has better clinical results in the short term, we should pay more attention to the long-term prosthesis survival rate regardless of the surgical technique used for total hip replacement. Indeed, proper placement of the prosthesis and maximum protection of the periarticular stabilization system reduces the rate of prosthetic dislocation, increases satisfaction in the short term, and contributes to long-term prosthetic survival.
